# Supplementary material for: Dicer promotes genome stability via the bromodomain transcriptional co-activator BRD4
Source: Nat Commun. 2022 Feb 22;13:1001. doi: 10.1038/s41467-022-28554-8 (PMC8863982; doi:10.1038/s41467-022-28554-8)
Supplement: Supplementary file 1 — Supplementary Information [file 41467_2022_28554_MOESM1_ESM.pdf]

Supplementary Information File

"Dicer promotes genome stability via the bromodomain transcriptional co-activator BRD4"

Gutbrod MJ, Roche B, Steinberg JI, Lakhani AA, Chang K, Schorn AJ, Martienssen RA.

List of Supplementary Figures

Figure S1 - Inducible deletion of *Dicer1* in mouse embryonic stem cells.

Figure S2 - Additional chromosomal phenotypes in *Dicer1*$^{-/-}$ mutants.

Figure S3 - *Dicer1*$^{-/-}$ mESCs have an apoptosis signature and low levels of major satellite transcript degradation products.

Figure S4 - Histone H3 Lysine 9 methylation is largely retained in the absence of *Dicer1*.

Figure S5 - *Dicer1*$^{-/-}$ mESCs retain H3K9me2/3 chromatin at transposable elements and satellite repeats, but lose H3K27me3.

Figure S6 - Screening and validation of *Ezh2* and *Brd4*/*Elp3* as enhancers and suppressors of *Dicer1*$^{-/-}$ viability defects.

Figure S7 - siRNA and small molecule inhibitors do not affect proliferation of wild type mESCs.

Figure S8 - BRD4 binding at genes does not change upon *Dicer1* mutation and BRD4 and ELP3 co-regulate genes and major satellite transcripts.

Figure S9 - Classes of suppressors interact and suppress chromosomal defects of *Dicer1*$^{-/-}$ mESCs.

Figure S10 - *bdf1Δ* and *bdf2Δ* in *S. pombe* are synthetic lethal via bromodomain 1 (BD1), but suppress *dcr1*Δ via BD2.

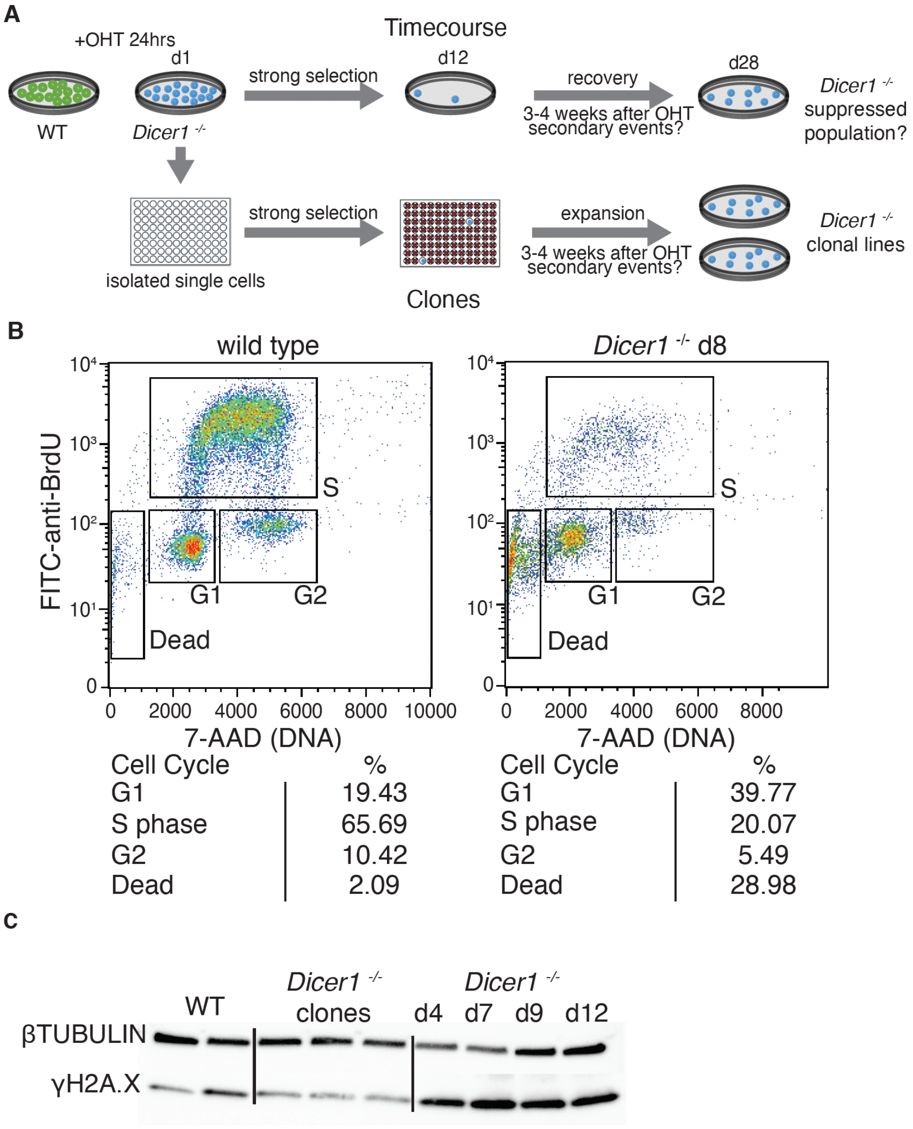

Figure S1. Inducible deletion of *Dicer1* in mouse embryonic stem cells.

(A) *Dicer1*$^{-/-}$ timecourse samples were derived from *Dicer1 flx/flx* mESCs that express a tamoxifen-inducible Cre recombinase by treatment with hydroxytamoxifen at day 0 (d0). Induction generated a population of *Dicer1*$^{-/-}$ cells that had lost both RNase III domains. These cells were grown for a period of 3-4 weeks in 2i media and resulted in stalled proliferation and apoptosis peaking at days 8-12 (d8-12) after induction. *Dicer1*$^{-/-}$ clones were derived from cells isolated immediately after induction of *Dicer1* mutation (d1). After 3-4 weeks in culture in 96 well plates, a small fraction of clones achieved a proliferation rate high enough to grow as a clonal cell line. After 4 weeks, timecourse populations were dominated by rare wild-type cells without rearrangement that were strongly selected for viability.

(B) Flow-cytometry analysis of the cell cycle in wild type and *Dicer1*$^{-/-}$ ES cells on day 8 after tamoxifen-induced deletion (*Dicer1*$^{-/-}$ d8). Cells were sorted into G1, S and G2 phase by BrdU incorporation and DNA content.

(C) DNA damage was assessed by Western blot using antibodies against γH2A.X in the *Dicer1*$^{-/-}$ timecourse and clones. Source Data and full-size blots are provided in the Source Data file.

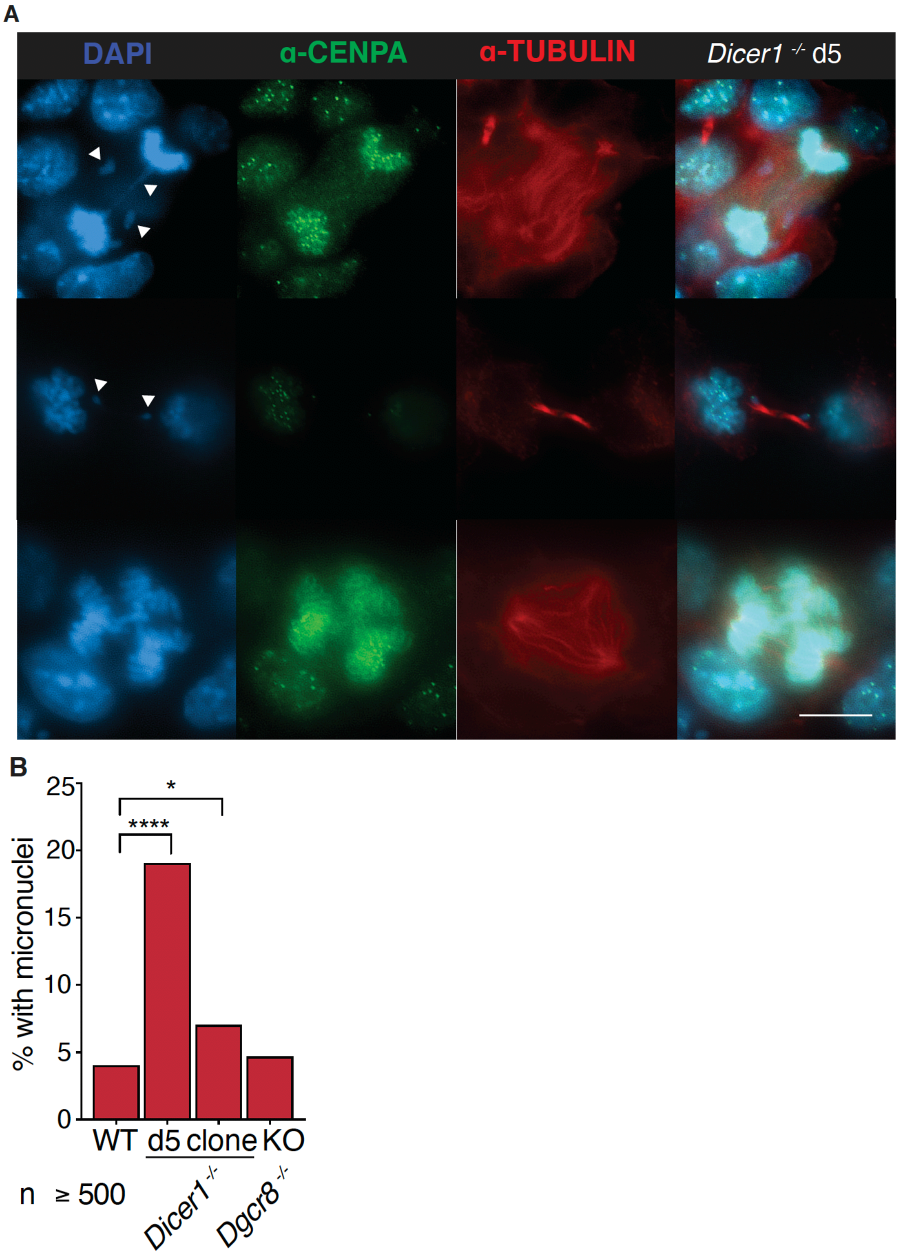

Figure S2. Additional chromosomal phenotypes in *Dicer1*$^{-/-}$ mutants.

(A) Immunofluorescence of CENPA (green) and tubulin (red) with DAPI staining (blue) shows CENPA incorporation in lagging chromosomes (top) and micronuclei (middle) and shows the underlying cytoskeletal structure of the tripolar mitoses observed (bottom). (Scale bar = 10 μm).

(B) Quantification of micronuclei at interphase (**** - p< 0.0001, * - 0.01 >p> 0.05 - Fisher's exact test, two-sided). Scale bar = 10 microns (n=500 cells examined over 2 independent experiments).

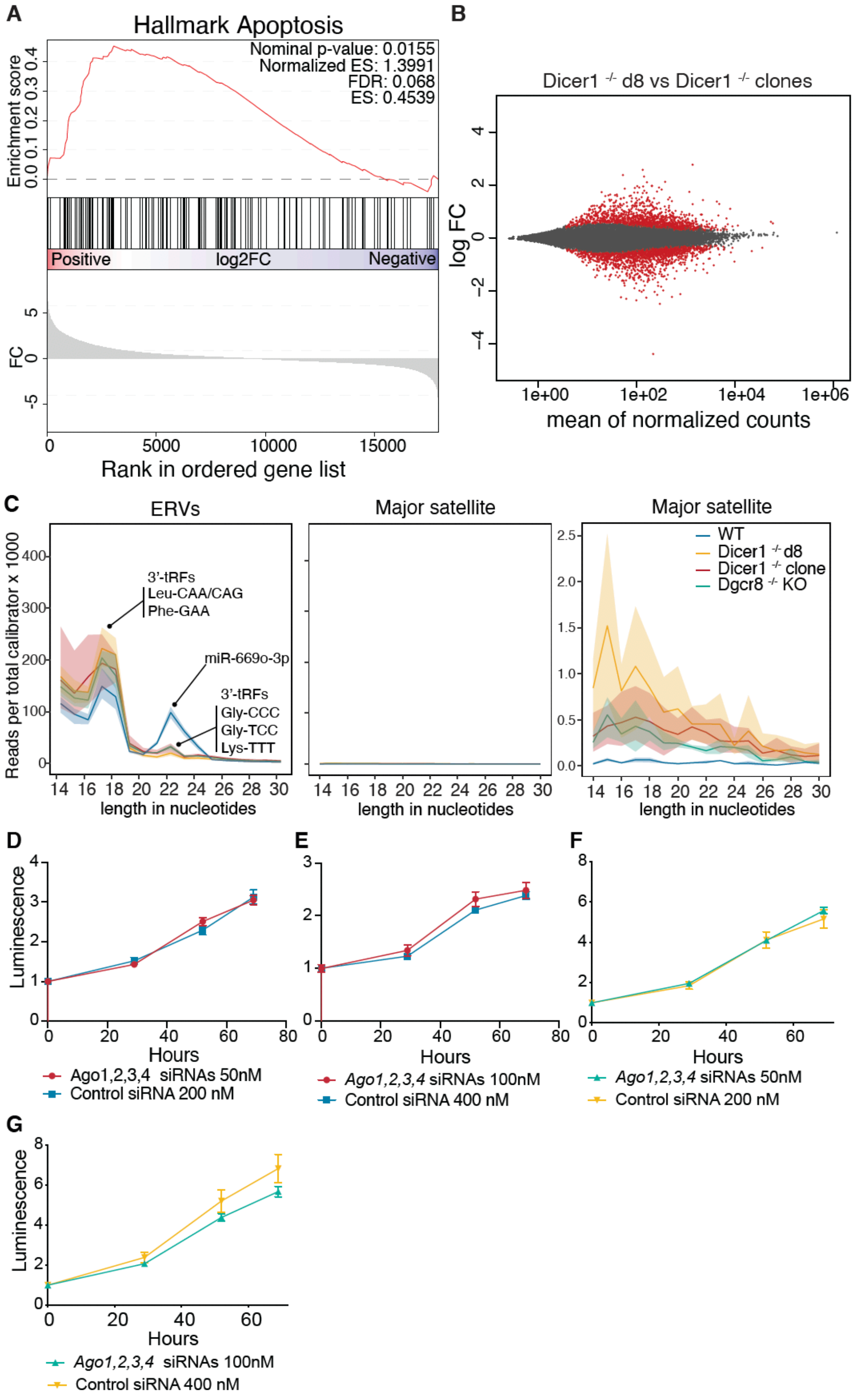

A
Hallmark Apoptosis
Nominal p-value: 0.0155
Normalized ES: 1.3991
FDR: 0.068
ES: 0.4539
Enrichment score
Positive
log2FC
Negative
FC
Rank in ordered gene list
B
Dicer1 -/- d8 vs Dicer1 -/- clones
log FC
mean of normalized counts
C
ERVs
3'-tRFs
Leu-CAA/CAG
Phe-GAA
miR-669o-3p
3'-tRFs
Gly-CCC
Gly-TCC
Lys-TTT
Reads per total calibrator x 1000
length in nucleotides
Major satellite
Major satellite
WT
Dicer1 -/- d8
Dicer1 -/- clone
Dgcr8 -/- KO
D
Luminescence
Hours
Ago1,2,3,4 siRNAs 50nM
Control siRNA 200 nM
E
Hours
Ago1,2,3,4 siRNAs 100nM
Control siRNA 400 nM
F
Hours
Ago1,2,3,4 siRNAs 50nM
Control siRNA 200 nM
G
Luminescence
Hours
Ago1,2,3,4 siRNAs 100nM
Control siRNA 400 nM

Figure S3. *Dicer1*$^{-/-}$ mESCs have an apoptosis signature and low levels of major satellite transcript degradation products.

(A) Hallmark Gene Set Enrichment analysis (GSEA) demonstrates an upregulation of apoptosis promoting factors in *Dicer1*$^{-/-}$ clones relative to wild type. The enrichment score (ES) reflects the overrepresentation of a gene set at the extremes of the ranked list of genes. The nominal p-value is calculated by a Kolmogorov-Smirnov test (two-sided) versus null distribution (n=1000 permutations), and adjusted for multiple hypothesis testing by the False Discovery Rate (FDR) method (cf. GSEA User Guide).

(B) Differential expression analysis identifies significant difference in transcripts altered in *Dicer1*$^{-/-}$ cells 8 days after deletion (d8) compared to mutant clones that have undergone selection for viability.

(C) Small RNA-seq identifies low levels of degradation products from the major satellite transcripts in *Dicer1*$^{-/-}$ clones. Results are plotted as counts normalized to calibrator spike-in abundance to account for the loss of microRNAs and shaded error bands indicate the range of replicate values (N=2 independent experiments). The right panel is a zoomed in plot of the center panel with an appropriate scale.

(D-G) A MT-like proliferation and viability assay shows siRNA treatments targeting all Argonaute proteins simultaneously have little effect on *Dicer1*$^{-/-}$ timecourse days 4-6 (D and E) or wild type (F and G) mESCs. One representative experiment is plotted (N=3 biological replicates), standard error bars may be smaller than points and represent SEM. The difference between control and Ago1,2,3,4 siRNAs was non-significant (p-value>0.05, two-sided t-test). Source Data are provided in the Source Data file.

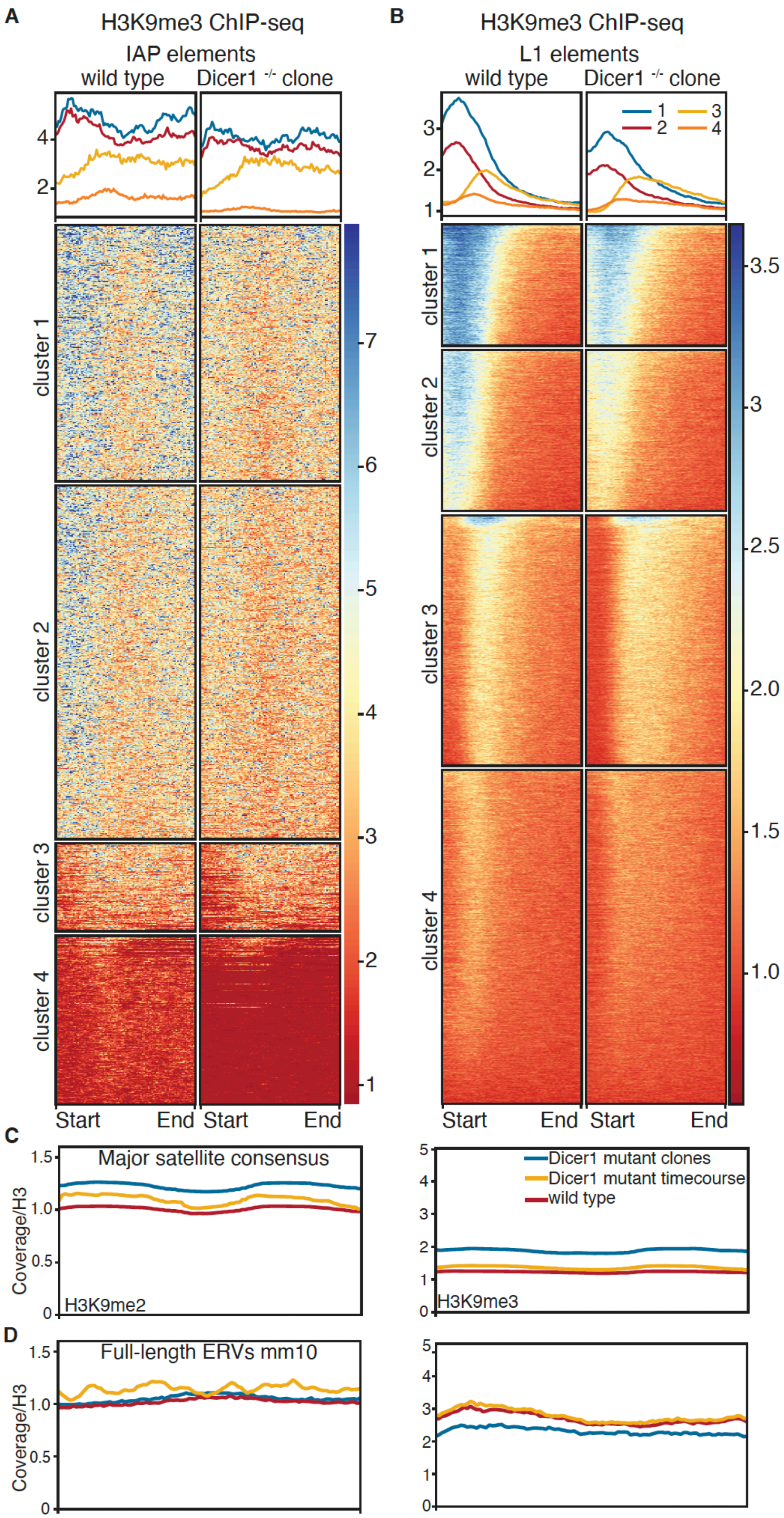
A
H3K9me3 ChIP-seq
IAP elements
wild type
Dicer1 -/- clone
cluster 1
cluster 2
cluster 3
cluster 4
Start
End
Start
End
B
H3K9me3 ChIP-seq
L1 elements
wild type
Dicer1 -/- clone
cluster 1
cluster 2
cluster 3
cluster 4
Start
End
Start
End
C
Major satellite consensus
Coverage/H3
H3K9me2
Dicer1 mutant clones
Dicer1 mutant timecourse
wild type
H3K9me3
D
Full-length ERVs mm10
Coverage/H3

Figure S4. Histone H3 Lysine 9 methylation is largely retained in the absence of *Dicer1*.

(A and B) Clustered heatmaps of H3K9me3 normalized to input and histone H3 over full length IAP (A) and LINE1 (B) transposable elements. Some subclasses of LINE1 elements have reduced H3K9me3 in *Dicer1*$^{-/-}$ day 8 cells, but most TEs are unaffected.

(C and D) ChIP-seq reads from H3K9me2 and H3K9me3 normalized to input and histone H3 in wild type, *Dicer1*$^{-/-}$ day 8, and *Dicer1*$^{-/-}$ clones were aligned to a 2x consensus sequence of the major satellite (C) and full length ERVs (D). H3K9me2 and 3 were largely retained in the absence of *Dicer1*.

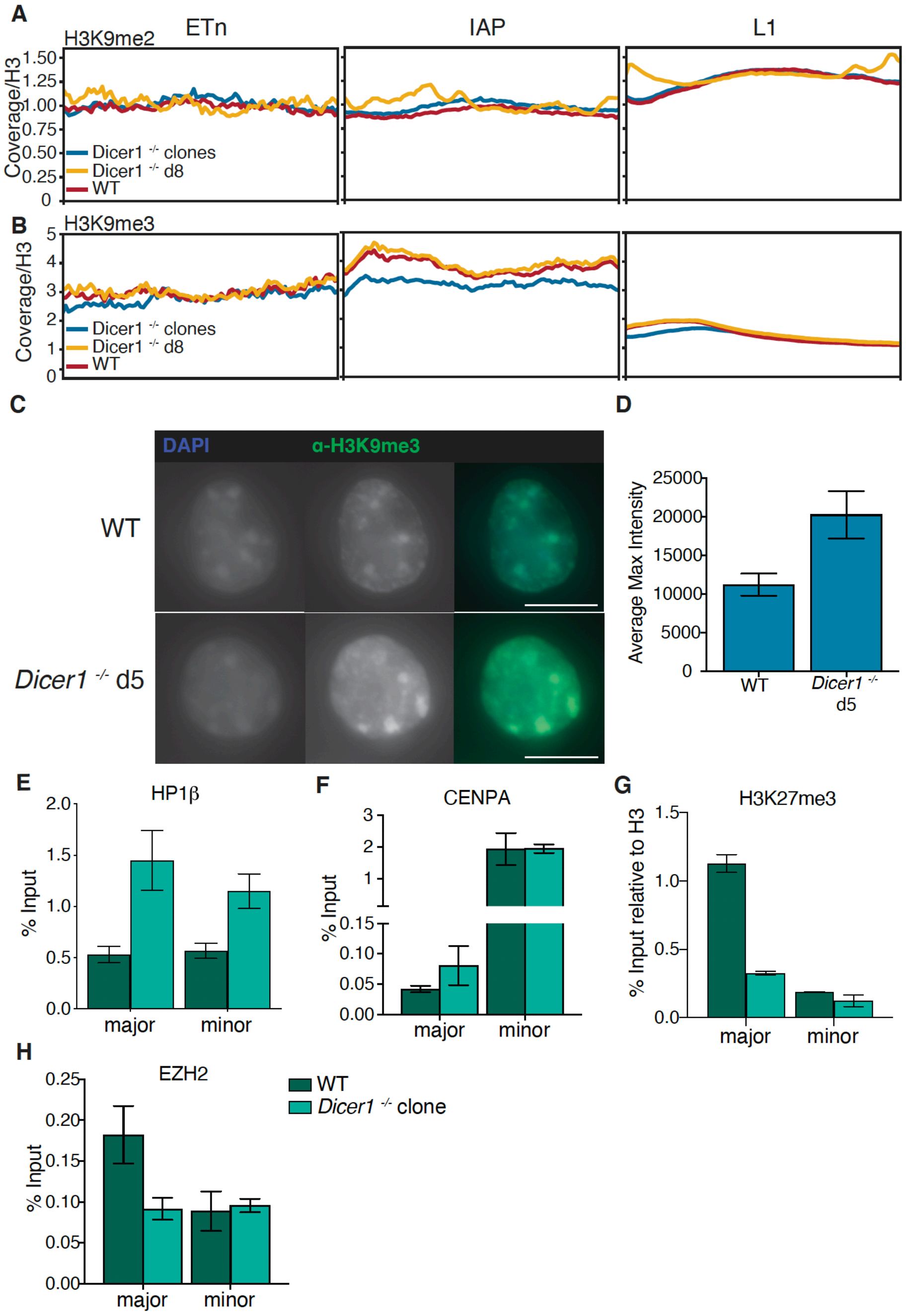

Figure S5. *Dicer1*$^{-/-}$ mESCs retain H3K9me2/3 chromatin at transposable elements and satellite repeats, but lose H3K27me3.

(A and B) The ChIP-seq coverage of H3K9me2 (A) and H3K9me3 (B) normalized to input and H3 control are plotted for wild type, *Dicer1*$^{-/-}$ timecourse day 8, and *Dicer1*$^{-/-}$ clonal line samples across all full length ETn elements (left), full length IAP elements (center), and L1 elements in the mm10 genome assembly.

(C and D) Immunofluorescence of H3K9me3 in uninduced wild type (C) and *Dicer1*$^{-/-}$ timecourse day 5 cells (D) showed a global increase in H3K9me3 in *Dicer1*$^{-/-}$

concentrated at chromocenters (scale bar = 10 μm). This increase was quantified as the average max intensity of collapsed Z-stacks acquired with the same exposure from multiple independent slides and experiments (n=50 cells for each of 5 independent biological replicates, p-value=0.0012, two-sided t-test). Error bars represent SD.

(E, F, G, H) ChIP-qPCR reveals the gain of HP1β in *Dicer1*$^{-/-}$ clonal lines at the major and minor satellite repeat loci (E), the maintenance of centromeric identity as measured by CENPA incorporation (F), and the loss of H3K27me3 (G) and EZH2 binding (H). Results are plotted as percent input. N=2 independent biological replicates; error bars represent SEM. Source Data are provided in the Source Data file.

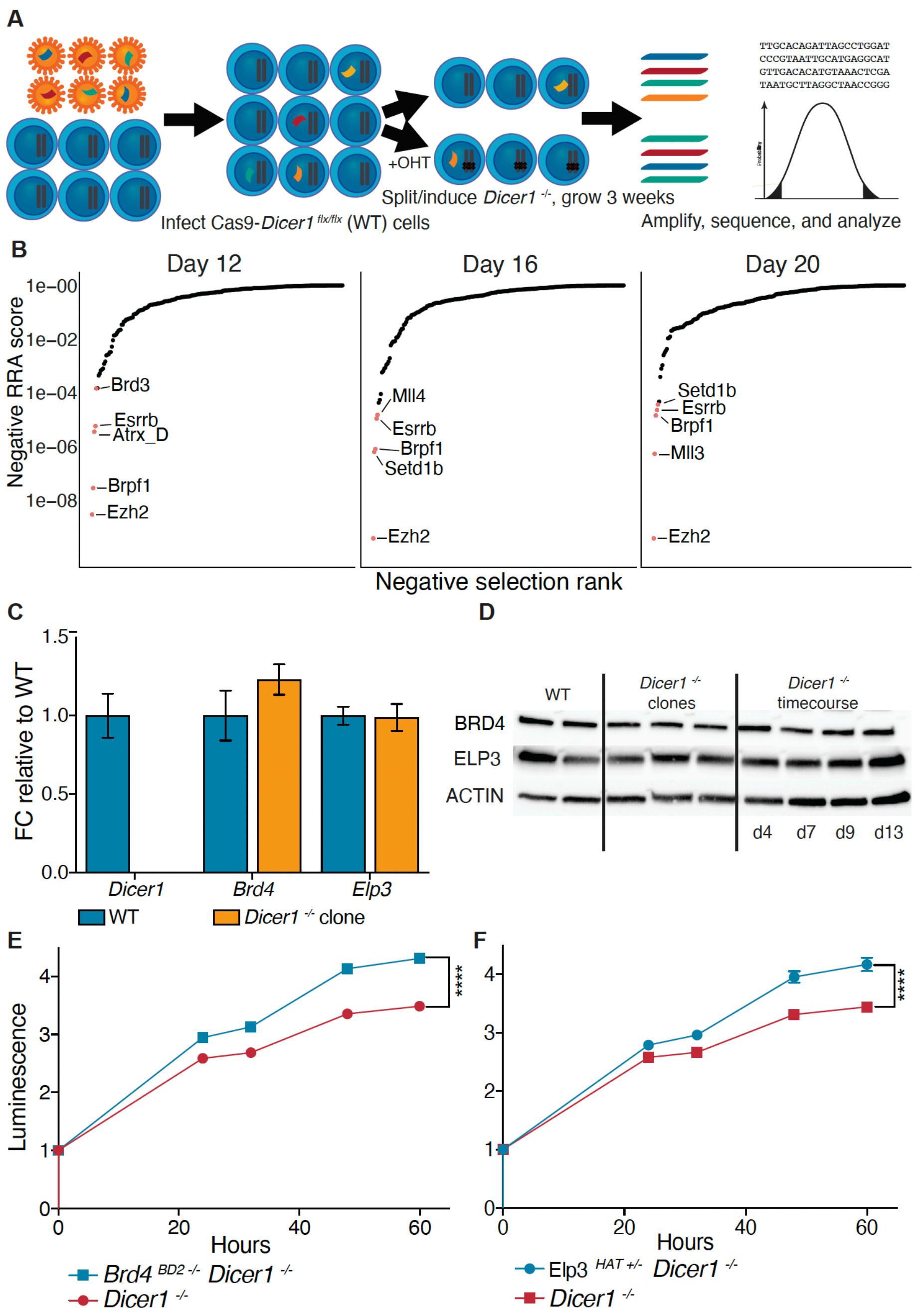

Figure S6. Screening and validation of *Ezh2* and *Brd4*/*Elp3* as enhancers and suppressors of *Dicer1*$^{-/-}$ viability defects.

(A) A CRISPR-Cas9 genetic screen was performed by transducing a *Dicer1 flx/flx* cell line that was constitutively expressing Cas9 with a domain-focused library of sgRNAs targeting chromatin modifiers in six replicate experiments. The transduced cells were split into two populations – (1). *Dicer1 flx/flx* untreated wild type replicates and (2). *Dicer1*$^{-/-}$ replicates that were treated with hydroxytamoxifen to induce recombination at the RNase III domains of both *Dicer1* alleles. The independent replicate populations were grown for a period of three weeks and genomic DNA was collected every 4 days

when the populations were split. The integrated sgRNA sequences were amplified from the population and subjected to next generation sequencing to determine the relative abundances in each replicate.

(B) The results of the CRISPR-Cas9 genetic screen are plotted according to the negative RRA score, which ranks genes based on the performance of all sgRNAs targeting them, and the ranking of genes as being negatively selected. The results at the timepoints following the phenotypic selection of *Dicer1* mutation are shown.

(C and D) The relative amounts of *Brd4* or *Elp3* do not change in *Dicer1*$^{-/-}$ day 8 at either the RNA level by RT-qPCR fold change calculated with normalization to *Actb* and uninduced wild type samples (C) (N=3 independent biological replicates, error bars represent SD) or the protein level as determined by Western blot (D). Therefore, they are very unlikely to be targets of miRNA. Source Data and full-size blots are provided in the Source Data file.

(E and F) A MT-like proliferation and viability assay during days 4-7 of the *Dicer1*$^{-/-}$ timecourse shows a suppression of the proliferation defect of *Dicer1* by 3 independent clonal mutations in the second bromodomain of *Brd4* (E) or the histone acetyltransferase domains of *Elp3* (F). One representative experiment is plotted, error bars represent SEM; standard error bars may be smaller than points, **** - p-value < 0.0001, two-sided t-test of final timepoints of replicate experiments (N=9 independent biological experiments).

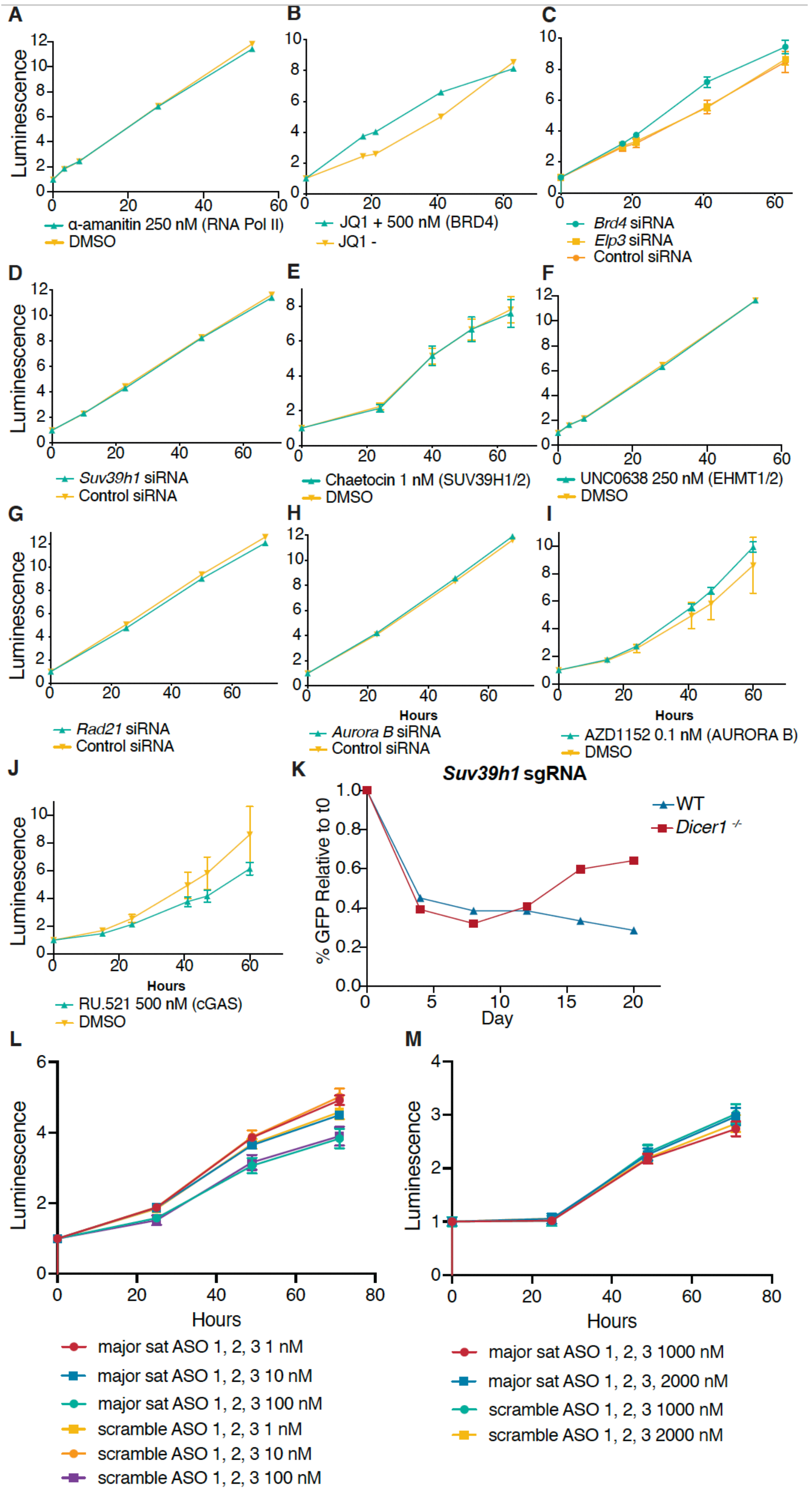
A
Luminescence
α-amanitin 250 nM (RNA Pol II)
DMSO
B
JQ1 + 500 nM (BRD4)
JQ1 -
C
Brd4 siRNA
Elp3 siRNA
Control siRNA
D
Luminescence
Suv39h1 siRNA
Control siRNA
E
Chaetocin 1 nM (SUV39H1/2)
DMSO
F
UNC0638 250 nM (EHMT1/2)
DMSO
G
Luminescence
Rad21 siRNA
Control siRNA
H
Hours
Aurora B siRNA
Control siRNA
I
Hours
AZD1152 0.1 nM (AURORA B)
DMSO
J
Luminescence
Hours
RU.521 500 nM (cGAS)
DMSO
K
Suv39h1 sgRNA
% GFP Relative to t0
Day
WT
Dicer1 -/-
L
Luminescence
Hours
major sat ASO 1, 2, 3 1 nM
major sat ASO 1, 2, 3 10 nM
major sat ASO 1, 2, 3 100 nM
scramble ASO 1, 2, 3 1 nM
scramble ASO 1, 2, 3 10 nM
scramble ASO 1, 2, 3 100 nM
M
Luminescence
Hours
major sat ASO 1, 2, 3 1000 nM
major sat ASO 1, 2, 3, 2000 nM
scramble ASO 1, 2, 3 1000 nM
scramble ASO 1, 2, 3 2000 nM

Figure S7. siRNA and small molecule inhibitors do not affect proliferation of wild type mESCs.

(A-J) A MT-like proliferation and viability assay shows siRNA and small molecule inhibitor treatments have little effect on wild type mESCs by targeting RNA Pol II with α-amanitin (A), *Brd4* with JQ1 (B), *Brd4* or *Elp3* with siRNAs (C), *Suv39h1* with siRNAs (D), *Suv39h1/2* with the inhibitor chaetocin (E), *Ehmt1/2* with the inhibitor UNC0638 (F), *Rad21* with siRNAs (G), *Aurora B* with siRNAs (H), *Aurora B* with the inhibitor AZD1152 (I), or cGAS with the inhibitor RU.521 (J). One representative experiment is plotted, error bars represent SEM, standard error bars may be smaller than points (N=3 independent biological experiments; non-significant using a two-sided t-test of final timepoints).

(K) Individual sgRNAs targeting the first exon of *Suv39h1* were introduced with eGFP reporter genes into *Dicer1*$^{-/-}$ cells, and GFP positive cells were counted at the same three timepoints after tamoxifen-induced deletion of *Dicer1* (upper panels) (N=3 independent biological replicates).

(L-M) An MT-like proliferation and viability assay shows that targeting the major satellite repeat with ASOs across a range of concentrations or with ASOs targeting different strands does not suppress the *Dicer1*$^{-/-}$ viability and proliferation defects. One representative experiment is plotted, error bars represent SEM, standard error bars may be smaller than points (N=3 independent biological experiments). Source Data are provided in the Source Data file.

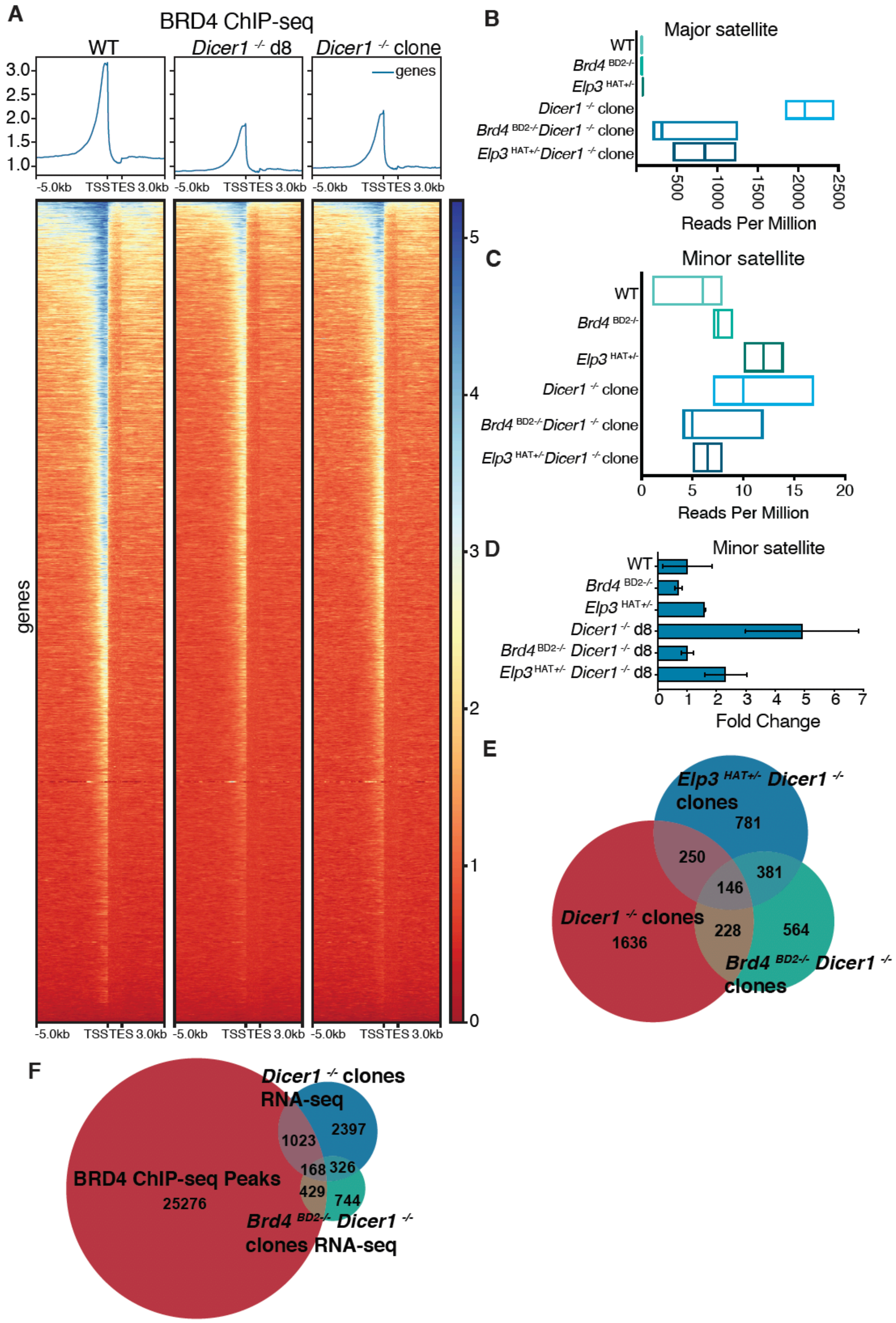
A
BRD4 ChIP-seq
WT
Dicer1 -/- d8
Dicer1 -/- clone
genes
3.0
2.5
2.0
1.5
1.0
-5.0kb
TSS
TES
3.0kb
genes
5
4
3
2
1
0
B
Major satellite
WT
Brd4 BD2-/-
Elp3 HAT+/-
Dicer1 -/- clone
Brd4 BD2-/- Dicer1 -/- clone
Elp3 HAT+/- Dicer1 -/- clone
500
1000
1500
2000
2500
Reads Per Million
C
Minor satellite
WT
Brd4 BD2-/-
Elp3 HAT+/-
Dicer1 -/- clone
Brd4 BD2-/- Dicer1 -/- clone
Elp3 HAT+/- Dicer1 -/- clone
0
5
10
15
20
Reads Per Million
D
Minor satellite
WT
Brd4 BD2-/-
Elp3 HAT+/-
Dicer1 -/- d8
Brd4 BD2-/- Dicer1 -/- d8
Elp3 HAT+/- Dicer1 -/- d8
0
1
2
3
4
5
6
7
Fold Change
E
Elp3 HAT+/- Dicer1 -/- clones
781
250
381
146
Dicer1 -/- clones
1636
228
564
Brd4 BD2-/- Dicer1 -/- clones
F
Dicer1 -/- clones RNA-seq
2397
1023
168
326
BRD4 ChIP-seq Peaks
25276
429
744
Brd4 BD2-/- Dicer1 -/- clones RNA-seq

Figure S8. BRD4 binding at genes does not change upon *Dicer1* mutation and BRD4 and ELP3 co-regulate genes and major satellite transcripts.

(A) The binding profile of BRD4 at all UCSC RefSeq Genes is plotted by normalized coverage over the transcriptional units from 5 kb upstream to 3 kb downstream. This analysis showed a general reduction in binding of BRD4 to promoter regions of genes in *Dicer1*$^{-/-}$ conditions.

(B and C) Normalized reads per million of the major satellite transcript shows the substantial reduction in abundance in *Brd4* or *Elp3* double mutants with *Dicer1* (B) while the minor satellite transcript levels remain relatively stable across all conditions. The line in each box represents the median. N=3 independent biological experiments; N=4 for *Brd4* mutants. Source Data are provided in the Source Data file.

(D) RT-qPCR confirmed an accumulation in *Dicer1*$^{-/-}$ mESCs of transcripts from the minor satellite (p=0.087, one-sided t-test), which was reduced in *Brd4*$^{BD2-/-}$ or *Elp3*$^{HAT+/-}$ doubles or *Dicer1* clones. Fold change was calculated relative to *Actb* and uninduced wild type controls (error bars are SEM, N=2 independent biological experiments). Source Data are provided in the Source Data file.

(E) An intersection of significantly differentially expressed transcripts upregulated in *Dicer1*$^{-/-}$ mESCs and downregulated in *Brd4*$^{BD2-/-}$ and *Elp3*$^{HAT+/-}$ double mutants demonstrate co-regulation of the majority of these transcripts by BRD4 and ELP3.

(F) An intersection of significantly differentially expressed transcripts in *Brd4*$^{BD2-/-}$ *Dicer1*$^{-/-}$ clonal lines with BRD4 ChIP-seq peaks within 10 kb of the locus identified in *Dicer1*$^{-/-}$. Importantly, all 146 co-regulated transcripts (which included the major satellite transcript) identified in panel D were included in the 168 peaks, but only the major satellite had dramatically increased levels of BRD4 ChIP in *Dicer1*$^{-/-}$ cells (Fig. 4A).

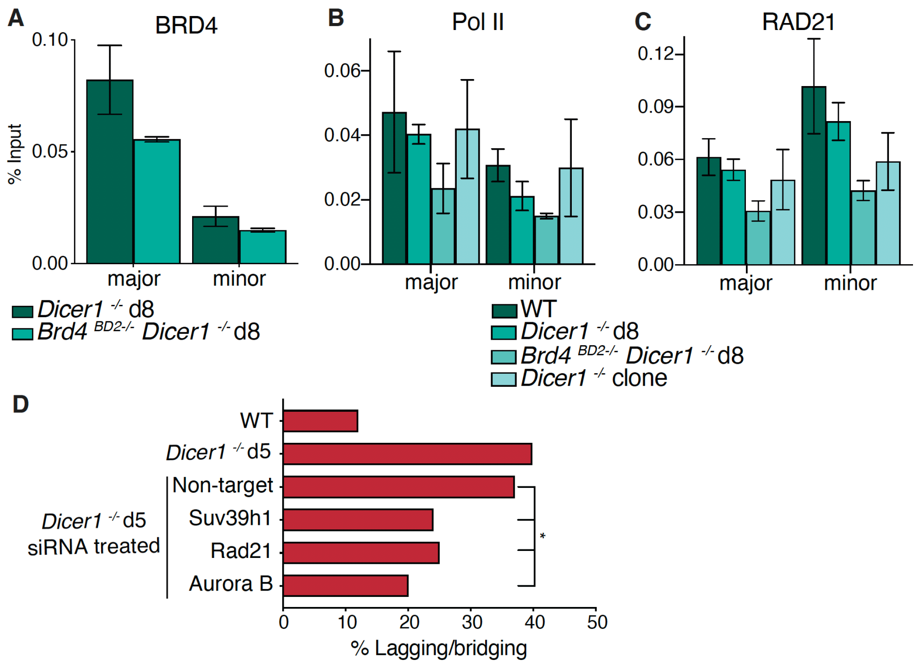

Figure S9. Classes of suppressors interact and suppress chromosomal defects of *Dicer1*$^{-/-}$ mESCs.

(A) ChIP-qPCR at the major and minor satellite repeats showed a decrease in BRD4 occupancy at satellite loci in *Brd4*$^{BD2-/-}$ *Dicer1*$^{-/-}$ timecourse cells compared to *Dicer1*$^{-/-}$ timecourse cells. The results are plotted as percent input (error bars represent SEM, N=2 independent biological replicates).

(B and C) ChIP-qPCR at the major and minor satellite repeats uncovered a reduction in active RNA polymerase II (Ser2/5 phosphorylated) (B) and RAD21 (C) in *Brd4*$^{BD2-/-}$

Dicer1$^{-/-}$ cells compared to *Dicer1*$^{-/-}$ cells 5 days after *Dicer1* deletion (d5). The results are plotted as percent input. Error bars represent SEM, N=2 independent biological replicates.

(D) Chromosomal defects are reduced in *Dicer1*$^{-/-}$ day 5 cells treated with siRNAs (n=100 cells over 2 independent biological experiments, * - p-value between 0.01 and 0.05, Fisher's exact test, two-sided). Source Data are provided in the Source Data file.

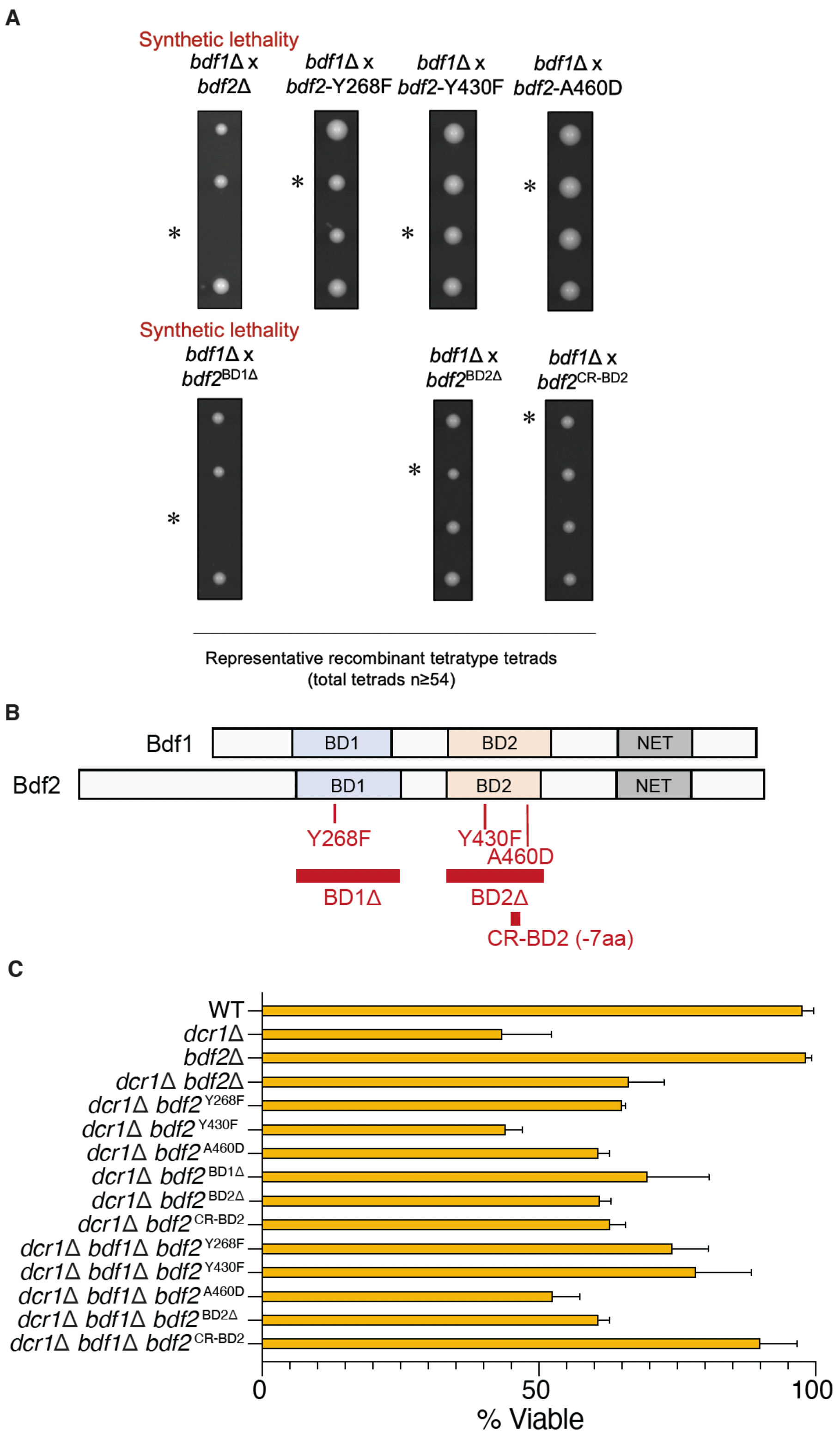

Figure S10. *bdf1Δ* and *bdf2Δ* in *S. pombe* are synthetic lethal via bromodomain 1 (BD1), but suppress *dcr1*Δ via BD2.

(A) Representative tetrads and quantification of viabilities in a *bdf1*Δ x *bdf2*Δ cross. The *bdf1*Δ*bdf2*Δ double-mutant is inviable, as determined from analysis of N=54 informative tetrads. Likewise, the *bdf1*Δ*bdf2*$^{BD1\Delta}$ mutant is inviable, while other combinations of mutants were fully viable and displayed no phenotype. For each tetrad, the star (*) indicates the double-mutant from representative recombinant tetrads (non-parental tetratype).

(B) Schematic of Bdf2 mutants used in the tetrad analysis experiments.

(C) Viability at G_0-entry (24h) in wild-type, *dcr1*Δ, and viable double and triple-mutants combinations of the Bdf1/Bdf2^{BRD4} genes. *dcr1*Δ*bdf2*$^{BD2\Delta}$ and *dcr1*Δ*bdf2*^{CRBD2} displayed suppression of the loss of viability of *dcr1*Δ similarly to that of *dcr1*Δ*bdf2*Δ. The strongest suppressor is the triple-mutant *dcr1*Δ*bdf1*Δ*bdf2*^{CRBD2}, which displayed wild-type viability. Error bars represent the SD from the mean, N=2 independent biological experiments (N=4 for wild-type, *dcr1*Δ, *bdf2*Δ, *dcr1*Δ*bdf2*Δ and *dcr1*Δ*bdf1*Δ*bdf2*^{CRBD2}). Source Data are provided in the Source Data file.
