# Supplementary material for: Dicer promotes genome stability via the bromodomain transcriptional co-activator BRD4
Source: Nat Commun. 2022 Feb 22;13:1001. doi: 10.1038/s41467-022-28554-8 (PMC8863982; doi:10.1038/s41467-022-28554-8)
Supplement: Supplementary file 8 — Reporting Summary [file 41467_2022_28554_MOESM8_ESM.pdf]

Corresponding author(s): R.A. Martienssen

Last updated by author(s): Dec 2, 2021

Reporting Summary

Nature Portfolio wishes to improve the reproducibility of the work that we publish. This form provides structure for consistency and transparency in reporting. For further information on Nature Portfolio policies, see our Editorial Policies and the Editorial Policy Checklist.

Statistics

For all statistical analyses, confirm that the following items are present in the figure legend, table legend, main text, or Methods section.

n/a	Confirmed	
☐	☒	The exact sample size (*n*) for each experimental group/condition, given as a discrete number and unit of measurement
☐	☒	A statement on whether measurements were taken from distinct samples or whether the same sample was measured repeatedly
☐	☒	The statistical test(s) used AND whether they are one- or two-sided<br>*Only common tests should be described solely by name; describe more complex techniques in the Methods section.*
☐	☒	A description of all covariates tested
☐	☒	A description of any assumptions or corrections, such as tests of normality and adjustment for multiple comparisons
☐	☒	A full description of the statistical parameters including central tendency (e.g. means) or other basic estimates (e.g. regression coefficient) AND variation (e.g. standard deviation) or associated estimates of uncertainty (e.g. confidence intervals)
☐	☒	For null hypothesis testing, the test statistic (e.g. *F*, *t*, *r*) with confidence intervals, effect sizes, degrees of freedom and *P* value noted<br>*Give P values as exact values whenever suitable.*
☒	☐	For Bayesian analysis, information on the choice of priors and Markov chain Monte Carlo settings
☒	☐	For hierarchical and complex designs, identification of the appropriate level for tests and full reporting of outcomes
☒	☐	Estimates of effect sizes (e.g. Cohen's *d*, Pearson's *r*), indicating how they were calculated

Our web collection on statistics for biologists contains articles on many of the points above.

Software and code

Policy information about availability of computer code

Data collection

MAGeCK 0.4
Flexbar 3.5.0
Trimmomatic 0.4
Bowtie 1.3.0
Bowtie2 2.4.3
FastX toolkit 0.0.14
Cutadapt 3.4
PRINSEQ 0.20.4
UMI-tools 1.0.0
Samtools 1.12
Bamtools 2.5.0
Bedtools 2.29.2
Freebayes 1.3.4
SnpEff 4.3
deepTools 3.5
STAR 2.7.7
TEtranscripts 2.2.1
DESeq2 3.11
GSEA 4
MACS2 2.2.5
Manorm 1.1.4
ChIP-Enrich 3.11
FlowJo 10.5.3

	GraphPad Prism 8 and 9
Data analysis	All data analysis was done with software listed above. Custom pipelines executing the software were used, but no custom software or analysis tools were used.

For manuscripts utilizing custom algorithms or software that are central to the research but not yet described in published literature, software must be made available to editors and reviewers. We strongly encourage code deposition in a community repository (e.g. GitHub). See the Nature Portfolio guidelines for submitting code & software for further information.

Data

Policy information about availability of data

All manuscripts must include a data availability statement. This statement should provide the following information, where applicable:

- Accession codes, unique identifiers, or web links for publicly available datasets
- A description of any restrictions on data availability
- For clinical datasets or third party data, please ensure that the statement adheres to our policy

Source data are provided with this paper. The next-generation sequencing data (DNA-seq, RNA-seq, small RNA-seq and ChIP-seq) generated in this study have been deposited in the NCBI GEO database under accession code GSE172282 (BioProject: PRJNA722747). Analysis of next-generation sequencing data is detailed in the Methods. Custom pipelines executing the software were used, but no custom software or analysis tools were used. The genome version used in this manuscript is Mus musculus mm10 from UCSC. All oligonucleotides used in this study are provided in Supplementary Data 5. All S. pombe strains generated in this study are provided in Supplementary Data 4 and are available upon request, as per standard practice.

Field-specific reporting

Please select the one below that is the best fit for your research. If you are not sure, read the appropriate sections before making your selection.

☒ Life sciences ☐ Behavioural & social sciences ☐ Ecological, evolutionary & environmental sciences

For a reference copy of the document with all sections, see nature.com/documents/nr-reporting-summary-flat.pdf

Life sciences study design

All studies must disclose on these points even when the disclosure is negative.

Sample size	For RNA-seq and small RNA-seq sample sizes of three were chosen to account for variability within RNA isolation and library preparation as is standard in the field. Three biological replicates is the recommended minimum for RNA-seq experiments (cf. Conesa et al, 2016, "A survey of best practices for RNA-seq data analysis"), as well as per the current ENCORE project guidelines (as of December 2016, cf. https://encodeproject.org/about/experiment-guidelines/) which recommend a minimum of two or more biological replicates for RNA-seq. For ChIP-seq experiments, the current ENCODE guidelines recommend a minimum of two biological replicates, and it was noted that the use of more than two replicates did not significantly improve the number of identified targets (based on RNA pol II ChIP-seq), which is why we therefore chose a sample size of two, as is standard practice, to capture the variation observed at the chromatin level. For cellular assays all experiments were done with three independent biological replicates, as is standard in the field, to account for variability in cell culture and assays.
Data exclusions	There was no data excluded.
Replication	All experiments were performed in duplicate or triplicate, as specified, at different times to ensure all possible sources of variability were accounted for. All attempts at replication of the results were successful.
Randomization	Randomization is not relevant to our study as we do not have experimental setup requiring allocation into experimental groups.
Blinding	For chromosome segregation analysis of immunofluorescence images, the evaluator was blinded to the genotype or treatment of the cells being visually inspected for segregation defects, for data collection and analysis. Other experiments were performed, as much as is possible and in accordance to standard practices in genetics, in a blinded manner.

Reporting for specific materials, systems and methods

We require information from authors about some types of materials, experimental systems and methods used in many studies. Here, indicate whether each material, system or method listed is relevant to your study. If you are not sure if a list item applies to your research, read the appropriate section before selecting a response.

Materials & experimental systems

n/a	Involved in the study
	☒ Antibodies
	☒ Eukaryotic cell lines
☒	Palaeontology and archaeology
☒	Animals and other organisms
☒	Human research participants
☒	Clinical data
☒	Dual use research of concern

Methods

n/a	Involved in the study
	☒ ChIP-seq
	☒ Flow cytometry
☒	MRI-based neuroimaging

Antibodies

Antibodies used

For ChIP, immunofluorescence and Western blotting, the following primary antibodies were used: H3K9me3 (Abcam ab176916), H3K9me2 (Cell Signaling 4658s), HP1B (Cell Signaling 8676s), Pol II Ser2/5p (Cell Signaling 13546), BRD4 (Active Motif 39910), H3K27me3 (Cell Signaling 9733s), EZH2 (Cell Signaling 5246s), CENPA (Cell Signaling 2047), beta-tubulin (Cell Signaling 2146s), H3 (Cell Signaling 4620s), ELP3 (Cell Signaling 5728), gamma-H2AX (Cell Signaling 2577s), actin (Cell Signaling 3700s).
Antibodies were used at a 1:1000 dilution for Western blots, and 1:500 for immunofluorescence experiments. For Western blots the secondary antibody was HRP goat anti-rabbit (Abcam ab97051). For immunofluorescence, donkey anti-rabbit Alexa Fluor 488 (Thermo A-21206) and goat anti-rabbit Alexa Fluor 594 (Thermo A-11012) were used.

Validation

All primary antibodies have been previously validated for use in mouse samples as stated on each company's respective website. In detail, these validations are as follows:
H3K9me3 (ab176916) has been validated for use in mouse by Western blot, immunofluorescence, flow cytometry, dot blot and ChIP. The manufacturer's website lists 21 publications using this antibody.
H3K9me2 (Cell Signaling 4658s) has been validated for use in mouse by Western blot, immunofluorescence, flow cytometry, and ChIP. This antibody detects endogenous levels of histone H3 only when dimethylated on Lys-9, and has been confirmed not to cross-react with non-methylated, mono-methylated or tri-methylated H3K9. Furthermore, the antibody does not cross-react with H3K4me2, H3K27me2, H3K36me2 nor H4K20me2. The manufacturer's website lists 96 publications using this antibody.
HP1B (Cell Signaling 8676s) has been validated for use in mouse by Western blot, immunofluorescence, and ChIP. This antibody detects endogenous levels of HP1B and does not cross-react with other HP1 proteins, including HP1alpha and HP1gamma. The manufacturer's website lists 9 publications using this antibody.
Pol II Ser2/5p (Cell Signaling 13546) has been validated for use in mouse by Western blot, ChIP and Cut-and-Run. This antibody recognizes endogenous levels of Rpb1 with a dual-phosphorylated CTD (Ser2+Ser5) and does not cross react with Pol II CTD repeats that are singly phosphorylated at Ser2, Ser5 or Ser7. The manufacturer's website lists 2 publications using this antibody.
BRD4 (Active Motif 39910) has been validated for use in mouse by Western blot, immunohistochemistry and ChIP.
H3K27me3 (Cell Signaling 9733s) has been validated for use in mouse by Western blot, immunofluorescence, immunohistochemistry, ChIP and Cut-and-Run. This antibody recognizes endogenous levels of H3 trimethylated on Lys27 and does not cross-react with unmethylated H3K27, H3K27me1 nor H3K27me2. Furthermore, this antibody does not cross-react with H3K4me3, H3K9me3, H3K36me3 nor H4K20me3. The manufacturer's website lists 582 publications using this antibody.
EZH2 (Cell Signaling 5246s) has been validated for use in mouse by Western blot, immunofluorescence, immunohistochemistry, ChIP and Cut-and-Run. This antibody detects endogenous levels of EZH2 protein, and does not cross-react with EZH1. The manufacturer's website lists 414 publications using this antibody.
CENPA (Cell Signaling 2047) has been validated for use in mouse by Western blot and immunoprecipitation. The antibody detects endogenous levels of mouse CENP-A protein, and does not cross-react with other histone proteins including H3. The manufacturer's website lists 1 publication using this antibody.
Beta-tubulin (Cell Signaling 2146s) has been validated for use in mouse by Western blot, immunofluorescence, immunohistochemistry and immunoprecipitation. The antibody detects endogenous levels of beta-tubulin and does not cross-react with alpha-tubulin. The manufacturer's website lists 559 publications using this antibody.
H3 (Cell Signaling 4620s) has been validated for use in mouse and optimized for ChIP. This antibody detects endogenous levels of histone H3 and does not cross-react with other histones. The manufacturer's website lists 169 publications using this antibody.
ELP3 (Cell Signaling 5728) has been validated for use in mouse by Western blot.
gamma-H2AX (Cell Signaling 2577s) has been validated for use in mouse by Western blot and immunofluorescence. This antibody detects endogenous levels of H2AX only when phosphorylated at Ser139. The manufacturer's website lists 659 publications using this antibody.
actin (Cell Signaling 3700s) has been validated for use in mouse by Western blot, immunofluorescence and immunohistochemistry. This antibody recognizes endogenous levels of beta-actin and, due to the high sequence identity between beta-actin and cytoplasmic gamma-actin, may cross-react with cytoplasmic gamma-actin. This antibody does not cross-react with alpha-actin isoforms nor smooth muscle isoforms of gamma-actin. The manufacturer's website lists 2127 publications using this antibody.
Donkey anti-rabbit Alexa Fluor 488 (Thermo A-21206) has been validated for use in mouse by immunohistochemistry (74 publications), immunofluorescence (53 publications) and flow cytometry (8 publications). To minimize cross-reactivity, this secondary antibody has been cross-adsorbed against serum proteins, resulting in higher sensitivity and lower background.
Goat anti-rabbit Alexa Fluor 594 (Thermo A-11012) has been validated for use in mouse by immunofluorescence (35 publications). To minimize cross-reactivity, this secondary antibody has been cross-adsorbed against human IgG, human serum, mouse IgG, mouse serum and bovine serum, resulting in higher sensitivity and lower background.

Eukaryotic cell lines

Policy information about cell lines

Cell line source(s)	Lenti-X 293T cells were acquired from Takara (632180). Dicer1flx/flx mES cells were a gift from Edith Heard. Briefly, these mES cells were obtained by isolation from CreERT2-Dicer1flx/flx mice, which were obtained by crossing Dicer1flx/flx mice (Murchinson et al, 2005) to ROSA-CreERT2 mice (Vooijs et al, 2001).
Authentication	The genetic modifications to generate the Dicer1 mutations were assayed for by PCR. The genetic lesion leading to Dgcr8 knock-out was verified by PCR.
Mycoplasma contamination	All cell lines were tested routinely for mycoplasma contamination, and all cell lines tested negative for mycoplasma contamination.
Commonly misidentified lines (See ICLAC register)	No commonly misidentified cell lines were used in the study, as per the latest version of the ICLAC Registry of Misidentified Cell lines: version 11, released 8 June 2021.

ChIP-seq

Data deposition

☒ Confirm that both raw and final processed data have been deposited in a public database such as GEO.

☒ Confirm that you have deposited or provided access to graph files (e.g. BED files) for the called peaks.

Data access links *May remain private before publication.*	GEO accession: GSE172282 (public access)
Files in database submission	GSM5251798 wild type_H3K9_ChIP_input_1<br>GSM5251799 wild type_H3K9_ChIP_input_2<br>GSM5251800 Dicer timecourse_H3K9_ChIP_input_1<br>GSM5251801 Dicer timecourse_H3K9_ChIP_input_2<br>GSM5251802 Dicer clone_H3K9_ChIP_input_1<br>GSM5251803 Dicer clone_H3K9_ChIP_input_2<br>GSM5251804 wild type_H3_ChIP_1<br>GSM5251805 wild type_H3_ChIP_2<br>GSM5251806 Dicer timecourse_H3_ChIP_1<br>GSM5251807 Dicer timecourse_H3_ChIP_2<br>GSM5251808 Dicer clone_H3_ChIP_1<br>GSM5251809 Dicer clone_H3_ChIP_2<br>GSM5251810 wild type_me2_ChIP_1<br>GSM5251811 wild type_me2_ChIP_2<br>GSM5251812 Dicer timecourse_me2_ChIP_1<br>GSM5251813 Dicer timecourse_me2_ChIP_2<br>GSM5251814 Dicer clone_me2_ChIP_1<br>GSM5251815 Dicer clone_me2_ChIP_2<br>GSM5251816 wild type_me3_ChIP_1<br>GSM5251817 wild type_me3_ChIP_2<br>GSM5251818 Dicer timecourse_me3_ChIP_1<br>GSM5251819 Dicer timecourse_me3_ChIP_2<br>GSM5251820 Dicer clone_me3_ChIP_1<br>GSM5251821 Dicer clone_me3_ChIP_2<br>GSM5251786 wild type_BRD4_ChIP_input_1<br>GSM5251787 wild type_BRD4_ChIP_input_2<br>GSM5251788 Dicer clone_BRD4_ChIP_input_1<br>GSM5251789 Dicer clone_BRD4_ChIP_input_2<br>GSM5251790 Dicer timecourse_BRD4_ChIP_input_1<br>GSM5251791 Dicer timecourse_BRD4_ChIP_input_2<br>GSM5251792 wild type_BRD4_ChIP_1<br>GSM5251793 wild type_BRD4_ChIP_2<br>GSM5251794 Dicer clone_BRD4_ChIP_1<br>GSM5251795 Dicer clone_BRD4_ChIP_2<br>GSM5251796 Dicer timecourse_BRD4_ChIP_1<br>GSM5251797 Dicer timecourse_BRD4_ChIP_2
Genome browser session (e.g. UCSC)	No saved session.

Methodology

Replicates	For all ChIP-seq samples there were two replicates per condition and antibody (as well as input), as per ENCODE project guidelines. The agreement between replicates was high for all samples as verified by the multiBamSummary and plotCorrelation functions of the deepTools software package. Only peaks that were consistent between replicates were considered for downstream analysis.

Sequencing depth	GSM5251798 wild type_H3K9_ChIP_input_1 14543484 14338421 75bp PE<br>GSM5251799 wild type_H3K9_ChIP_input_2 17617223 17377629 75bp PE<br>GSM5251800 Dicer timecourse_H3K9_ChIP_input_1 13418190 13228994 75bp PE<br>GSM5251801 Dicer timecourse_H3K9_ChIP_input_2 16715648 16461570 75bp PE<br>GSM5251802 Dicer clone_H3K9_ChIP_input_1 13610635 13251314 75bp PE<br>GSM5251803 Dicer clone_H3K9_ChIP_input_2 21333633 21024295 75bp PE<br>GSM5251804 wild type_H3_ChIP_1 17701172 17495838 75bp PE<br>GSM5251805 wild type_H3_ChIP_2 14815466 14646570 75bp PE<br>GSM5251806 Dicer timecourse_H3_ChIP_1 14776303 14598987 75bp PE<br>GSM5251807 Dicer timecourse_H3_ChIP_2 11929281 11783744 75bp PE<br>GSM5251808 Dicer clone_H3_ChIP_1 16888962 16699806 75bp PE<br>GSM5251809 Dicer clone_H3_ChIP_2 11461921 11331255 75bp PE<br>GSM5251810 wild type_me2_ChIP_1 17849106 17556381 75bp PE<br>GSM5251811 wild type_me2_ChIP_2 20471005 20159846 75bp PE<br>GSM5251812 Dicer timecourse_me2_ChIP_1 15446000 15123179 75bp PE<br>GSM5251813 Dicer timecourse_me2_ChIP_2 13733309 13160630 75bp PE<br>GSM5251814 Dicer clone_me2_ChIP_1 18310301 17881840 75bp PE<br>GSM5251815 Dicer clone_me2_ChIP_2 19166410 18773499 75bp PE<br>GSM5251816 wild type_me3_ChIP_1 16455699 16202281 75bp PE<br>GSM5251817 wild type_me3_ChIP_2 17986965 17713563 75bp PE<br>GSM5251818 Dicer timecourse_me3_ChIP_1 14536328 14293571 75bp PE<br>GSM5251819 Dicer timecourse_me3_ChIP_2 25980082 25559205 75bp PE<br>GSM5251820 Dicer clone_me3_ChIP_1 18149085 17844180 75bp PE<br>GSM5251821 Dicer clone_me3_ChIP_2 16406406 16145544 75bp PE<br>GSM5251786 wild type_BRD4_ChIP_input_1 107817054 105897910 75bp PE<br>GSM5251787 wild type_BRD4_ChIP_input_2 107387637 105562047 75bp PE<br>GSM5251788 Dicer clone_BRD4_ChIP_input_1 51339494 50399981 75bp PE<br>GSM5251789 Dicer clone_BRD4_ChIP_input_2 17783034 17450491 75bp PE<br>GSM5251790 Dicer timecourse_BRD4_ChIP_input_1 23883906 23346518 75bp PE<br>GSM5251791 Dicer timecourse_BRD4_ChIP_input_2 35709565 34748978 75bp PE<br>GSM5251792 wild type_BRD4_ChIP_1 19121863 18666763 75bp PE<br>GSM5251793 wild type_BRD4_ChIP_2 23858930 23310175 75bp PE<br>GSM5251794 Dicer clone_BRD4_ChIP_1 11659724 11362401 75bp PE<br>GSM5251795 Dicer clone_BRD4_ChIP_2 19723521 19196903 75bp PE<br>GSM5251796 Dicer timecourse_BRD4_ChIP_1 30562673 29370729 75bp PE<br>GSM5251797 Dicer timecourse_BRD4_ChIP_2 18233923 17675965 75bp PE
Antibodies	For ChIP the following primary antibodies were used: H3K9me3 (Abcam ab176916), H3K9me2 (Cell Signaling 4658s), BRD4 (Active Motif 39910), and H3 (Cell Signaling 4620s). These antibodies were validated for ChIP as stated on the manufacturer's website (cf above, "Antibody validation").
Peak calling parameters	Reads were trimmed for adapters and quality with Trimmomatic. Bowtie2 was used for mapping with default settings and --dovetail -X 600 -k 5 parameters. The resulting SAM files were converted, sorted, and indexed with Samtools. Normalized coverage was calculated for library size for each sample and a relative normalized coverage was calculated for IP over input using deepTools. Peaks were called with default settings with MACS2. Differential peaks were called with default settings with MANorm. Peaks were assigned to underlying or nearby features and a functional enrichment analysis of these features was performed with ChIP-Enrich using default parameters. For various analyses, both input and H3 antibody controls were used.
Data quality	All ChIP-seq results were validated with qPCR from 3 independent biological replicates. Only results with FDR=0.1 were considered.
Software	Reads were trimmed for adapters and quality with Trimmomatic. Bowtie2 was used for mapping with default settings and --dovetail -X 600 -k 5 parameters. The resulting SAM files were converted, sorted, and indexed with Samtools. Normalized coverage was calculated for library size for each sample and a relative normalized coverage was calculated for IP over input using deepTools.

Flow Cytometry

Plots

Confirm that:

☒ The axis labels state the marker and fluorochrome used (e.g. CD4-FITC).

☒ The axis scales are clearly visible. Include numbers along axes only for bottom left plot of group (a 'group' is an analysis of identical markers).

☒ All plots are contour plots with outliers or pseudocolor plots.

☒ A numerical value for number of cells or percentage (with statistics) is provided.

Methodology

Sample preparation	BrdU Flow Cytometry Cell Cycle Analysis was performed with a FITC BrdU Flow Kit (BD Pharmingen 559619). Cells were cultured normally, then dosed with an appropriate amount of BrdU for at least 1 hour prior to harvest. Cells were washed in PBS, run over a strainer cap flow cytometry tube, and analyzed. The Flow Kit protocol was followed as directed and the Bio-Rad SE3 Cell Sorter was used.

Instrument	The Bio-Rad SE3 Cell Sorter was used.
Software	Bio-Rad flow cytometry software was used for acquisition. FlowJo software (version 10.5.3) was used for analysis.
Cell population abundance	Cells were not sorted for use after flow cytometry experiments.
Gating strategy	For BrdU analysis, FSC/SSC was used to first gate live cells which are a small distinct population in mESCs. Then the various cell cycle stage gates were generated according to the FITC BrdU Flow Kit manual. The gating strategy is provided in Fig S1. For single sgRNA experiments, the same initial gating strategy was used to gate live cells. EGFP-positive cells were then gated using a simple scatter plot strategy to identify only clearly EGFP-positive cells in the positive gate, excluding all cells in-between positive and negative.

☒ Tick this box to confirm that a figure exemplifying the gating strategy is provided in the Supplementary Information.
